# Work Engagement as a Moderating Factor between Positive Attitude toward Smart Working and Job and Life Satisfaction

**DOI:** 10.3390/ejihpe12070057

**Published:** 2022-07-11

**Authors:** Andrea Zammitti, Angela Russo, Paola Magnano, Maria Guarnera

**Affiliations:** 1 Department of Educational Sciences, University of Catania, 95121 Catania, Italy; angela.russo@phd.unict.it; 2 Faculty of Human and Social Sciences, Kore University, Cittadella Universitaria, 94100 Enna, Italy; paola.magnano@unikore.it (P.M.); maria.guarnera@unikore.it (M.G.)

**Keywords:** smart working, attitude, work engagement, job satisfaction, life satisfaction

## Abstract

Attitude toward smart working reflects feelings of favorableness towards this object; attitudes influence intentions, which in turn guide behaviors. Recent research confirms the positive influence that attitude toward smart working has on expected usage of it. Despite a direct influence, other factors could interact in the context of opportunities for ICT usage for teleworkers; among these factors, work engagement stands out. In turn, work engagement influences the perception of job satisfaction and life satisfaction. Considering that literature suggests that among the antecedents of work engagement are attitudes, the present study analyzes the role of positive attitude towards smart working on work engagement, and consequently on job satisfaction and on life satisfaction, hypothesizing that work engagement could mediate between positive attitude towards smart working and job and life satisfaction. The participants were 342 workers (115 males and 227 females) in private and public organizations, aged 24 to 66 years. The results showed that a positive attitude towards smart working, along with work engagement as a mediator, positively influences job satisfaction and life satisfaction. This means that employers and human resources managers (HRM) can organize training sessions to enhance the positive attitude toward smart working and this can help workers feel more engaged and satisfied.

## 1. Introduction

The health emergency of Covid-19 had a huge impact on the spread of smart working, accelerating changes in work organization already underway as a result of the technological evolution known for more than twenty years. Smart working, remote working, WFH-Working from Home, agile work, teleworking, telecommuting, and e-working are some of the names typically used to describe these “non-conventional organisational models that are characterized by higher flexibility and autonomy in the choice of working spaces, time and tools, and that provides all employees of an organisation with the best working conditions to accomplish their tasks” [1] (p. 338). Unlike in previous years, the COVID-19 pandemic forced the introduction of smart working, denying the possibility to many employees and employers to choose between the traditional work forms and the remote ones. Furthermore, in the future, the multifaceted technological society could lead to an exponential increase in remote jobs. In this scenario, it is important to understand how to promote positive organizational attitudes, behaviors and well-being of workers.

Many studies have been conducted to explore the effect of smart working on workers’ well-being [2], exploring both positive outcomes, such as those associated with greater flexibility and productivity, and the negative outcomes frequently associated with technostress and the maintenance of work-life balance (for a review, see [3]). For example, the study of Felstead and Henseke [4] suggests that the greater spatial and temporal flexibility of smart working may be beneficial for workers’ organizational commitment, job satisfaction and job-related well-being. A study by Grant et al. [5] showed that smart workers’ well-being was linked to effective organizational communication, peer group support, relationships outside of working hours, and interactions with family members. A more recent study by Grant et al. [6] found that mental well-being was significantly correlated with work productivity, confidence in the organization, and better job management in terms of flexibility. Furthermore, the study of Zeike et al. [7] highlighted that the perception of having adequate digital leadership skills impacted workers’ levels of psychological well-being when choosing to introduce smart working in the organization. In line with this, during the pandemic period, Angelici and Profeta [8] show that the flexibility of smart working improves satisfaction with social life, free time, and life in general. Surprisingly, Angelici and Profeta’s results [8] show that the job satisfaction of 200 smart-workers (treated group), unlike that of 110 traditional workers (control group), increases even if they applied more effort; perhaps smart-workers seek to exchange more effort for more flexibility to maintain or even increase their job satisfaction. This suggests that workers had a positive attitude towards the new form of work organization.

Attitude toward smart working reflects a general evaluation or feeling of favorableness towards this object [9]. Attitudes are a summary evaluation or feeling of favorableness towards an object. Eagly and Chaiken [10] define an attitude as “a psychological tendency that is expressed by evaluating a particular entity with some degree of favor or disfavor” (p. 1). Attitudes have different characteristics [11]: they can have a positive, negative, or neutral valence; they can vary in intensity; they may be more or less resistant to change. Attitudes influence intentions, which in turn guide behaviors [12,13]. Therefore, if a worker has a positive attitude towards telecommuting, then it is more likely that he intends to use it [14,15] in the context of his/her work. In consonance with this, a more recent study by Malik et al. [9] confirms the positive influence that attitude towards smart working has on expected usage of it. Such direct influence showed a smaller magnitude, highlighting that other personal, job and firm factors could interact in the broader context of opportunities for ICT usage for teleworkers [9].

Among these factors, work engagement emerges as one of the main areas of interest that has recently been most discussed in the literature deepening smart working [3]. Work engagement can be defined as “a positive, fulfilling, work-related state of mind that is characterized by vigor, dedication and absorption“ [16] (p. 702). According to Schaufeli et al. [17], a work-engaged individual has high levels of vigor, which concerns energy, mental resilience, and persistence. Moreover, work-engaged individuals feel dedicated, experiencing a sense of significance, enthusiasm, inspiration, and pride in their job. Finally, being engaged means working in a state of complete concentration on one’s work, namely absorption. In other words, work engagement can be seen as a persistent and pervasive affective-motivational state that is not focused on any particular object, event, individual or behavior, but is linked to wider work-related wellbeing.

It is widely known that workers’ engagement influences the perception of job satisfaction (e.g., [18,19,20]) and life satisfaction [21,22]. On an individual level, job satisfaction and life satisfaction are closely related and mutually influence each other [23]. On the one hand, job satisfaction is defined as a worker’s positive attitude resulting from the appraisal of job experience [24]. There is no unanimous consent about the working characteristics that affect job satisfaction [25]. Seashore et al. [26] argued that the influencing factors of job satisfaction could be divided into two groups, i.e., the environmental factors and the individual factors [27]. Among the latter, according to Linz’s [28] it is possible to trace a positive attitude towards work. Despite this, Susanty and Miradipta’s study [11] showed that attitude toward work has a positive but not significant effect on job satisfaction. On the other hand, life satisfaction is defined as “a global assessment of a person’s quality of life according to his chosen criteria” [29] (p. 478). It is a cognitive-judgmental process [30], resulting from positive feelings in several dimensions of people’s lives [31] and linked to many work-related antecedents, regarding need satisfaction, mindful activity, and job-related tensions (for a review, see [31]). Among these, De Cuyper et al. [32] identified a significant relationship between life satisfaction and work engagement. Subsequently, Ferreira et al. [33] underlined that work engagement can be related to other aspects of workers’ lives besides their job and organizational roles, because working life is a part of the wider personal life, as demonstrated by studies on work-life balance.

Literature has shown different individual and organizational antecedents of work engagement, such as job characteristics [19,20], psychological capital (namely self-efficacy, hope, resilience, and optimism; [34]), authentic leadership (namely self-awareness, relational transparency, balanced processing, and internalized moral perspective [35]); leaders’ autonomy-support climate [36] and perceived organizational support [19,20]. Noteworthy, Judge and Kammeyer-Mueller [37] suggested that engagement, as motivational energy, is likely to be influenced by attitudes.

To the best of the authors’ knowledge, to date, no study has yet explored the role of the positive attitude towards smart working and work engagement on job satisfaction and life satisfaction.

### Aims of the Study

Following the framework of positive psychology [38,39] and the need to investigate and find effective positive resources for employees within changing world of work, the purpose of this paper is to investigate the role of positive attitude towards smart working on work engagement, and consequently on job satisfaction and on life satisfaction. Accordingly, in this study, we hypothesized that:

**Hypothesis** **H1.** *Work engagement would mediate the relationship between a positive attitude toward smart working and job satisfaction*.

**Hypothesis** **H2.** *Work engagement would mediate the relationship between a positive attitude towards smart working and life satisfaction*.

## 2. Method

### 2.1. Procedure and Participants

An a priori power analysis was conducted using G*Power software, version 3.1.9.7 [40,41], to evaluate the minimum sample size to predict Life Satisfaction and Job Satisfaction with two predictor variables. The parameters indicated in the literature were maintained to carry out this analysis: a medium effect size of 0.15 (Effect size f^2^ = 0.15) with α = 0.05 and minimum Power (1 − β) = 0.95 [42]. The analysis revealed that a minimum total sample size of 107 participants was necessary.

After this, we created a survey that included the measures described in the next paragraph. Participants voluntarily participated in the research and were recruited from the general population, through convenience sampling. The survey was preceded by the following indications: “Dear participant, we are conducting research about smart working and life and organizational satisfaction. We invite you to participate in this study by completing an online protocol. In the beginning, some socio-demographic variables will be collected, such as age, gender, and educational qualification. You will then be asked questions relating to the research topics and you will be asked to indicate your personal agreement or disagreement”. There were also indications about the research manager, the data treatment—in an anonymous and aggregated form–and the lack of risks to personal well-being. All these precautions fall within the indications of the ethical code of the Italian Association of Psychology [43]. The protocol was sent to some companies that have collaborated with the university in past research and advertised through social networks (Facebook and LinkedIn).

The following inclusion criteria were considered: have a minimum age of 16 (the minimum age to be able to work in Italy), be resident in Italy and be a worker in an organization present in the Italian territory. To check that the participants had these characteristics in the personal data section, in addition to their age, there was a question relating to the respondent’s residence and the type of organization in which he worked (private organization, public organization or work as a freelancer). This allowed us to eliminate all those who did not meet the requirements for this research.

Participants were also asked to provide a code consisting of the first two letters of their name, the first two letters of their surname and their day of birth. This allowed us to eliminate any double administrations.

Three hundred and seventy-one workers filled out our survey; 18 administrations were discarded as incomplete. Another 11 administrations were eliminated as they did not respect the criterion of being workers in private or public organizations. At the end, the participants deemed suitable for the present research were 342, 115 males (33.6%) and 227 females (66.4%), aged between 24 and 66 years (M = 48.04; SD = 10.37). Most of them had a university degree (145, 42.4%), and the others had a high school diploma (100, 29.2%) or a post-graduate degree (93, 27.2%); a very small part had a middle school license (4; 1.2%). Most of the participants worked for a public organization (271, 79.2%), while the remaining worked for a private organization (71, 20.8%). Descriptive characteristics of the sample are found in Table 1.

Based on the priori analysis, our sample is appropriate for testing the proposed model.

### 2.2. Measures

The survey used in the present research was divided into the following sections:

#### 2.2.1. Biographical Data

They were required to indicate their age, gender, educational attainment, and typology of organization (public or private).

#### 2.2.2. Measurement of Positive Attitude towards Smart Working

To evaluate Positive attitude towards smart working, we used 4 items that asked to indicate the degree of agreement/disagreement on a 5-point Likert scale (from 1 = absolutely disagree, to 5 = absolutely agree). The items–created ad hoc for the purposes of the study–were statements relating to a positive attitude towards smart working, seen as a tool for organizational improvement (sample item is “I believe that smart working can improve some streamlining processes in my organization”). Cronbach’s alpha of the scale for the study sample was 0.71.

#### 2.2.3. Measurement of Life Satisfaction

To evaluate Life Satisfaction the Italian version of Satisfaction With Life Scale (SWLS; [30,44]) was used. This is a scale composed of 5 items on a 7-point Likert scale (from 1 = Strongly disagree, to 7 = Strongly agree). The participant is asked to indicate how well each statements fits themselves. A sample item is “If I could live my life over, I would change almost nothing”. The Cronbach’s alpha for the study sample was 0.93.

#### 2.2.4. Measurement of Work Engagement

Work Engagement was evaluated with Italian short version of the Utrecht Work Engagement Scale (UWES-9; [45]). The participants responded to each item on a scale from 0 (never) to 6 (always). The 9 items refer to experiences related to work (sample item is “When I get up in the morning, I feel like going to work”). The Cronbach’s alpha for the study sample was 0.94.

#### 2.2.5. Measurement of Job Satisfaction

Job Satisfaction was measured using the Organizational Satisfaction Questionnaire (QSO; [46]). The QSO consists of 20 items. This questionnaire measures job satisfaction by three job satisfaction facets. The items indicate different aspects of the work and participants are requested to indicate their degree of satisfaction. We used 14 items to evaluate the aspect of *Satisfaction with the work itself* (sample item is “Autonomy level of my job”). The answers are given on a seven-point Likert scale (from 1 = completely unsatisfied, to 7 = completely satisfied). Cronbach’s alpha of the scale for the study sample was 0.95.

### 2.3. Data Analysis

Preliminary analyses (descriptive statistics, Cronbach’s alpha coefficients, and correlations) were run by SPSS. Structural Equation Modeling was implemented by AMOS [47].

To assess our hypotheses, we started by specifying a Structural Equation Model (SEM) for the full sample. The literature points out that a model is considered good if it respects the following parameters: if the ratio of the χ^2^ per degrees of freedom (χ^2^/df) is lower than 3, if the Incremental Fit Index (IFI) and the comparative fit index (CFI) values are approximately 0.90 or above [48], and if the root mean square error of approximation (RMSEA) is about 0.08 or less [49]. The 95% confidence intervals for the direct, simple indirect, and total indirect mediation effects were estimated using bootstrapping on 5000 samples.

To investigate whether the estimated effects were similar for female and male we used a multiple-group model. To do this, measurement invariance was tested by inspecting changes in fit across the configural, weak invariance, and structural models. Chen [50] recommends that the assumption of invariance across models is considered acceptable if ΔCFI < 0.01 and ΔRMSEA < 0.015.

## 3. Results

### 3.1. Preliminary Analyses

In the first step, descriptive statistics of measures, Cronbach’s alpha coefficients and correlations between measures and age were computed.

Descriptive statistics of measures are found in Table 2. The normality of the data distribution was shown by Shapiro–Wilk test; a significant level of this statistic (*p* < 0.05) indicated deviations from normality. All dimensions did not have a normal distribution. No changes to the data have been made.

Due to the non-normality of the data, the correlations were calculated using Spearman’s correlation coefficient (non-parametric method). Cronbach’s alpha coefficients appear to be significantly above the minimum threshold for Cronbach’s alpha coefficient of 0.60 [51]. Significant positive correlations were found between all dimensions. There was no correlation with age. Results are shown in Table 3.

### 3.2. CFA of the Measures

An assessment of the properties of the measurement model was conducted before testing the structural model. We ran a CFA, using AMOS, according to Harman’s single-factor test to determine the extent to which common-method variance was a problem. We compared the hypothesized model and a model with one factor, in which all the items loaded on a unique factor.

In the first step, the specified model provided a not very good fit for the data, with a χ^2^ per degrees of freedom of 3.89, a IFI of 0.846, a CFI of 0.846, RMSEA of 0.092 and AIC of 1922,766. Looking for a better-fitting model resulted in the inclusion of three error covariances (between items UWES1 and UWES2, UWES8 and UWES9, and QSO5 and QSO6). This gave us a model with good fit indices: χ^2^/df = 2.85, IFI = 0.902, CFI = 0.902, RMSEA = 0.074, AIC = 1442.729.

These errors covariances are explicable in relation to content. The first pair of items refers to high levels of energy and mental resilience while working [46] and uses the term “energy” in the item UWES1 and “strong and vigorous” in the item UWES2. The error covariance may suggest a redundancy of the items, as energy, strong and vigorous could be considered synonymous. The second pair of items refers to absorption, which is being fully concentrated on one’s work [46]. The terms used in the items are “I am immersed” in item UWES8 or “I get carried away”. Again, these terms suggest redundancy. The last couple of items refer to the dimension of Satisfaction with the work itself and refer to “Information and internal communication” (QSO5) and “Planning and control of activities” (QSO6). Both refer to internal aspects of the organization which, unlike the other items on the scale, have no reference to personal relationships or individual aspects. In this case, it appears to be an overlap of content between these two items.

The model with one factor, in which all the items loaded on a unique factor, showed the following fit indices: χ^2^/df = 7.81, IFI = 0.636, CFI = 0.634, RMSEA = 0.141 and AIC = 3735.710.

The hypothesized model provided a better fit for the data in all the CFA fit measures. The differences were significant according to a comparison of the models’ χ^2^ values and degrees of freedom: ∆χ^2^ (6) = 2304.981 (*p* < 0.000). According to these results, we found no evidence for common-method bias in the data.

### 3.3. Structural Equation Model

**Figure 1:**
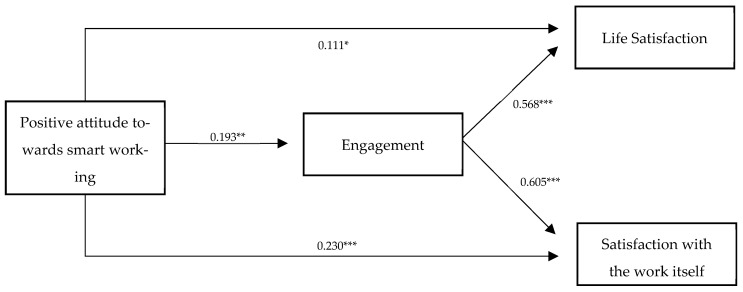
The structural model. * *p* < 0.05, ** *p* < 0.01, *** *p* < 0.001.

We applied structural equation model analysis to test our hypotheses. The main fit indices suggested that the model fit the data adequately: χ^2^/df = 2.86, IFI = 0.902, CFI = 0.901, RMSEA = 0.074. Figure 1 presents the final model. All the relationships between the variables are indicated by standardized beta.

Positive attitude towards smart working had a significant and positive direct effect on Satisfaction with the work itself (β = 0.23, *p* < 0.001), Life Satisfaction (β = 0.15, *p* < 0.05), and Engagement (β = 0.13, *p* < 0.005). Engagement had a significant and positive direct effect on Satisfaction with the work itself (β = 0.89, *p* < 0.001), and Life Satisfaction (β = 1.09, *p* < 0.001). Estimates for pathways are reported in Table 4.

On this model, using multiple-group SEM, we tested gender invariance to confirm that the model was adequate for both female and male. Analyses confirmed a weak factor invariance (ΔCFI = 0.001, and ΔRMSEA = 0.000) and structural invariance (ΔCFI = 0.000, and ΔRMSEA = 0.000).

We also tested the invariance between private and public organization, to confirm that the model was adequate for both typologies. Also, in this case, analyses confirmed a weak factor invariance (ΔCFI = 0.001, and ΔRMSEA = 0.001) and structural invariance (ΔCFI = 0.000, and ΔRMSEA = 0.000).

## 4. Discussion

In our complex and technological society, the need to understand how to drive positive psychological outcomes such as job satisfaction and life satisfaction emerges. Moving from the framework of positive psychology [39] and positive organizational behavior [52], the aim of this paper was to demonstrate that a positive attitude towards smart working, along with work engagement, could positively influence job satisfaction and life satisfaction.

Literature has already widely demonstrated that work engagement–the positive, fulfilling state of mind connected to the dedication and absorption of one’s physical, cognitive, and emotional energy in work activities [16,53]–is linked to several well-being outcomes, such as job satisfaction and life satisfaction (for a meta-analysis, see [54]). Besides, both engagement and satisfaction are likely to be influenced by attitudes, which are general evaluations of an object as it relates to personal values [28,37,55]. Therefore, even the attitude towards smart working could influence work engagement and satisfaction.

Moving from these premises, we have assumed that work engagement would have mediated the relationship between a positive attitude towards smart working and the two positive outcomes, namely job satisfaction (H1) and life satisfaction (H2). The results confirm both our hypotheses.

Regarding the first hypothesis, the results show that a positive attitude toward smart working has a direct relationship with work engagement and job satisfaction; moreover, work engagement mediates the relationship between a positive attitude towards smart working and job satisfaction. The results of this study reinforce the assumption of Linz [28] and the positive results already obtained regarding attitude toward work in general by Susanty and Miradipta [11], suggesting that attitude towards smart working had a positive and significant effect on job satisfaction. Probably, to assess attitudes, it is useful to evaluate them towards specific objects related to work, such as smart working. Moreover, consistent with this result, Felstead and Henseke [4] found that worker autonomy concerning the possibility of choosing whether or not to adhere to remote working practices acted as a mediator between the introduction of smart working in the company and job satisfaction.

Regarding the second hypothesis, the results indicate that a positive attitude toward smart working had a significant and positive influence on work engagement and life satisfaction; moreover, work engagement act as a mediator between the positive attitude towards smart working and life satisfaction. This is in line with the large existing literature (e.g., [21,22]), which exhibits that work engagement is related to increases in life satisfaction. Our innovative results regard the role of the positive attitude towards smart working in influencing not only work engagement, but also general life satisfaction. According to our study, having a positive attitude to smart working means believing that removing the constraints of space and time of work is a useful tool for organizational improvement or a promising way for a more efficient work organization that can improve some streamlining organizational processes (for example thought creating multidisciplinary workgroups) and that could bring positive transformations to the organizational culture. By developing a positive attitude towards smart working, workers can experience the implementation of smart working practices as a positive and desirable product of their commitment to creating sustainable work environments and feel more satisfied.

## 5. Conclusions

Public and private organizations must increasingly show the ability to translate the challenges of the changing and technological world of work into opportunities to create the human and social value capable of moving towards the Sustainable Development Goals of the 2030 Agenda.

On this basis, we introduced the concept of positive attitude towards smart working to further explore how the perception of smart working can influence not only vigor, dedication, and absorption to work, but also job satisfaction and general life satisfaction. The results of the present study show that a positive attitude toward smart working and people’s work engagement can influence both job satisfaction and life satisfaction.

Our study has some implications regarding the factors that could influence life and job satisfaction. In today’s complex digital world, among the other environmental and individual factors that are related to job and life satisfaction, a positive attitude towards smart working, as well as work engagement, could influence both life satisfaction and job satisfaction. Moreover, our study has some human resources management implications. Employers and human resources managers (HRM) can organize training sessions to enhance the positive attitude toward smart working and highlight the benefits of smart working to their employees. Implementing participatory human resources actions to support changes in working methods and procedures could solicit positive feelings and foster the willingness to change in workers. When smart working is seen as an opportunity to positively transform organizational culture and improve organizational streamlining processes, workers can feel more engaged and satisfied.

Future longitudinal research could test if the flexibility introduced by smart working may contribute to reduce gender gaps at work. Moreover, researchers could investigate whether other personal (such as gender, age, degree of education or personality factors) and contextual (such as the type of job or type of work-life balance benefit) variables influence the positive attitude toward smart working and, consequently, the satisfaction outcomes of personal and life satisfaction.

## 6. Limitations

This study has some limitations to be acknowledged. First, it is a cross-sectional study, and therefore it does not allow us to draw causal conclusions on the relationships between the variables. Second, it relies on self-report measures, which can lead to biased effects. Third, the convenience sampling and the snowball method to collect data limit the ability to generalize our findings to all workers. Finally, there could be a possible influence of other psychosocial factors on the variables under investigation, such as personality factors or type of work-life balance benefit; for example, a proactive personality is shown to be capable of predicting job satisfaction, both directly and indirectly through work engagement, in a group of teachers [56] and in a group of nurses [57].

## Figures and Tables

**Table 1:** Characteristics of the sample.

**Age**, M (SD)	**48.04 (10.37)**
**Gender**, n (%)	
Male	115 (33.6)
Female	227 (66.4)
**Educational attainment**, n (%)	
Middle school license	4 (1.2)
High school diploma	100 (29.2)
University degree	145 (42.4)
Post-graduate degree	93 (27.2)
**Type of organization** n (%)	
Public	271 (79.2)
Private	71 (20.8)

**Table 2:** Descriptive statistics.

**Positive attitude towards smart working** **M (DS), Shapiro–Wilk (*p value*)**	**3.44 (0.94), 0.96 (*0.000*)**
**Life Satisfaction** M (DS), Shapiro–Wilk (*p value*)	4.80 (1.42), 0.97 (*0.000*)
**Engagement** M (DS), Shapiro–Wilk (*p value*)	5.21 (1.21), 0.96 (*0.000*)
**Satisfaction with the work itself** M (DS), Shapiro–Wilk (*p value*)	4.49 (1.34), 0.98 (*0.002*)

**Table 3:** Bivariate correlations between model variables (Spearman’s rho).

	1	2	3	4	5
Age	1	−0.006	−0.058	−0.003	0.098
2. Positive attitude towards smart working		(0.71)	0.286 **	0.196 **	0.193 **
3. Satisfaction with the work itself			(0.95)	0.483 **	0.611 **
4. Life Satisfaction				(0.93)	0.522 **
5. Engagement					(0.94)

Note: ** *p* < 0.01. The Cronbach’s values are shown in brackets.

**Table 4:** Results of SEM analysis.

Link			B	SE	β
Positive attitude towards smart working	→	Engagement	0.133	0.045	0.193 **
Positive attitude towards smart working	→	Satisfaction with the work itself	0.230	0.054	0.230 ***
Positive attitude towards smart working	→	Life Satisfaction	0.146	0.071	0.111 *
Engagement	→	Satisfaction with the work itself	0.885	0.113	0.605 ***
Engagement	→	Life Satisfaction	1.09	0.139	0.568 ***

Note: B = unstandardized beta; SE = standard error; β = standardized beta. * *p*  < 0.05, ** *p* < 0.01, *** *p* < 0.001.

## Data Availability

The data are available from the corresponding author upon reasonable request.
